# Inhibition of the Fission Machinery Mitigates OPA1 Impairment in Adult Skeletal Muscles

**DOI:** 10.3390/cells8060597

**Published:** 2019-06-15

**Authors:** Vanina Romanello, Marco Scalabrin, Mattia Albiero, Bert Blaauw, Luca Scorrano, Marco Sandri

**Affiliations:** 1 Veneto Institute of Molecular Medicine, via Orus 2, 35129 Padua, Italy; marco.scalabrin94@gmail.com (M.S.); mattia.albiero@unipd.it (M.A.); bertblaauw@yahoo.com (B.B.); luca.scorrano@unipd.it (L.S.); 2 Department of Biomedical Science, University of Padua, via G. Colombo 3, 35100 Padua, Italy; 3 Myology Center, University of Padua, via G. Colombo 3, 35100 Padova, Italy; 4 Department of Medicine—DIMED, University of Padua, 35128 Padua, Italy; 5 Department of Biology, University of Padua, via U. Bassi 58B, 35121 Padua, Italy; 6 Department of Medicine, McGill University, Montreal, QC H3A 0G4, Canada

**Keywords:** mitochondrial fusion, fission, mitophagy, FGF21, muscle wasting, muscle dystrophy

## Abstract

The maintenance of muscle mass and its ability to function relies on a bioenergetic efficient mitochondrial network. This network is highly impacted by fusion and fission events. We have recently shown that the acute deletion of the fusion protein Opa1 induces muscle atrophy, systemic inflammatory response, precocious epithelial senescence, and premature death that are caused by muscle-dependent secretion of FGF21. However, both fusion and fission machinery are suppressed in aging sarcopenia, cancer cachexia, and chemotherapy-induced muscle wasting. We generated inducible muscle-specific Opa1 and Drp1 double-knockout mice to address the physiological relevance of the concomitant impairment of fusion and fission machinery in skeletal muscle. Here we show that acute ablation of Opa1 and Drp1 in adult muscle causes the accumulation of abnormal and dysfunctional mitochondria, as well as the inhibition of autophagy and mitophagy pathways. This ultimately results in ER stress, muscle loss, and the reduction of force generation. However, the simultaneous inhibition of the fission protein Drp1 when Opa1 is absent alleviates FGF21 induction, oxidative stress, denervation, and inflammation rescuing the lethal phenotype of Opa1 knockout mice, despite the presence of any muscle weakness. Thus, the simultaneous inhibition of fusion and fission processes mitigates the detrimental effects of unbalanced mitochondrial fusion and prevents the secretion of pro-senescence factors.

## 1. Introduction

Skeletal muscle is the most abundant tissue in the human body, functioning as a metabolic organ that demands an enormous amount of energy during contraction [1,2]. Mitochondrial ATP synthesis is critical for sustaining such an energetic request. Thus, a functional mitochondrial network is essential in this tissue. Skeletal muscle fibers are postmitotic cells that do not divide, and as a consequence, they cannot dilute dysfunctional mitochondria through cellular division. Therefore, muscles depend on the activation of the quality control mitochondrial pathways to maintain mitochondrial function [3]. There are several defensive strategies which are known as the mitochondria quality control pathways, and these pathways weaken the amount of mitochondrial damage. They include the activation of mitochondrial proteolytic systems, mitochondrial dynamics, and mitophagy, which is the autophagy–lysosome degradation of the mitochondrial fraction. Mitochondrial dynamics by alternating fusion and fission events allow the organelle to rapidly adapt its size and shape to the bioenergetics requirements of the cell. Optic atrophy 1 (Opa1) and mitofusin 1 and 2 (Mfn1/2) are the essential proteins that promote mitochondrial fusion to generate an interconnected network to optimize ATP production. The dynamic-related protein 1 (Drp1) and the mitochondrial fission factor (Mff) regulate mitochondrial fission. Segregating dysfunctional mitochondria from the network allows for their removal via mitophagy [3]. Alterations in mitochondrial content, shape, or function are closely associated with muscle wasting [3]. We and others have demonstrated that a failure in mitochondrial dynamics has detrimental consequences for the maintenance of muscle mass and function [3,4,5,6,7,8]. In muscles, the Drp1 inhibition causes muscle weakness and wasting that is consequent to an abnormal calcium homeostasis [8]. On the other hand, the overexpression of the fission proteins Drp1 and Fis1 in muscles causes muscle atrophy [5,7]. Conversely, overexpression of Opa1 protects from denervation-induced muscle loss and ameliorates muscle function in a mouse model of mitochondrial myopathy [9,10]. Muscle-specific ablation of both Mfn1 and Mfn2 induces muscle atrophy and abnormal muscle growth in mice [4]. Moreover, the specific deletion of Opa1 in muscle leads to oxidative stress, Endoplasmic Reticulum (ER) stress, and muscle atrophy [6]. Opa1-null muscles reverberate systemically, inducing metabolic alterations, inflammation, precocious senescence of different epithelial tissues and premature death via Fgf21 [6]. In fact, the deletion of both Opa1 and Fgf21 significantly reverts precocious aging and mortality [6]. However, sarcopenic muscles have alterations in both mitochondrial fusion and fission processes [3]. We provided evidence that, in humans, there are significant age-dependent decreases of the mitochondrial shaping proteins, Mfn1, Mfn2, Opa1, and Drp1 that correlate with muscle wasting [6]. Our data confirm previous observations indicating simultaneous reduction of mitochondrial fusion and fission in aged mice, rats, and humans [6,11,12,13,14]. Moreover, the concomitant decrease of Opa1 and Drp1 also occurs in cancer cachexia and chemotherapy-induced cachexia [15,16,17] as well as a model of myasthenia gravis [18]. However, the physiological role of the combined inhibition of mitochondrial fusion and fission factors in muscles has not yet been fully investigated. We generated tamoxifen-inducible muscle-specific Opa1/Drp1 double-knockout (DKO) to study in vivo the physiological relevance in muscle homeostasis of the abrogation of mitochondrial dynamics. Our results pointed to muscles that were characterized by the accumulation of abnormal mitochondria, ER stress, and the block of both autophagy and mitophagy bulk. The combined reduction of fusion and fission processes is a compensatory mechanism to mitigate muscle denervation, oxidative stress, muscle-derived Fgf21 release, and inflammation in aging sarcopenia, despite the consequences of muscle dysfunction.

## 2. Materials and Methods

### 2.1. Handling and Generation of Muscle-Specific Opa1/Drp1-Null Mice

All animals were handled by specialized personnel under the control of inspectors from the Veterinary Service of the Local Sanitary Service (ASL 16-Padua) who are the local officers from the Ministry of Health. The use of the animals and the experimental protocol followed were approved by the ethical committee and by the animal welfare coordinator of the OPBA from the University of Padua. All procedures are specified in the projects approved by the Italian Ministero Salute, Ufficio VI (authorization number: 1060/2015 PR), and were conducted in accordance with the relevant codes of practice for the care and use of animals for scientific purposes. Muscles were removed at various time periods and frozen in liquid nitrogen for subsequent analyses. 

To generate constitutive muscle-specific Opa1/Drp1 knockout animals, we used two genetic lines. One line was mice-bearing Opa1-floxed alleles (Opa1 ^fl/fl^) [19] that were then crossed with the other transgenic mouse line which expressed Cre Recombinase under the control of a Myosin Light Chain 1 fast promoter (MLC1f-Cre)-bearing heterozygous Dpr1-floxed alleles (MLC-Drp1^+/fl^) [8]. Cre-mediated recombination led to in-frame deletion of exon 2 encoding, the GTP-binding motif of the Drp1 gene, and to the deletion in *Opa1* gene of exons 2 and 3. The Opa1 deletion resulted in an aberrant exons 1–4 transcript with a stop codon after exon 1, producing a predicted 10 amino acid (aa) residual protein. No homozygous MLC-Drp1/Opa1 Cre-positive-floxed mice were obtained, suggesting embryonic lethality (Table S3, Supplementary Materials). 

A second knockout model with inducible muscle-specific inactivation of Opa1 and Dpr1 was obtained by crossing mice-bearing Drp1-floxed alleles (Drp1^fl/fl^) [8] with muscle-specific Opa1 transgenic mice carrying Cre-ER driven by human skeletal actin (HSA) promoter [6]. Tamoxifen-induced Cre LoxP recombination was activated by oral administration of tamoxifen-containing chow (Tam400/Cre ER Harlan) which was administered ad libitum for 5 weeks. Muscles were collected at different time points after the tamoxifen diet was finished. Cre-negative littermates, also receiving tamoxifen treatment, were used as controls. A minimum of 3 adult mice (3 to 5 months old) of the same sex and age of each genotype were used for each experiment. PCR genotyping was performed with the following primers:

DRP1 Fw: CAGCTGCACTGGCTTCATGACTC
DRP1 Rv: GTCAACTTGCCATAAACCAGAG
OPA1 Fw: CAGTGTTGATGACAGCTCAG
OPA1 Rv: CATCACACACTAGCTTACATTTGC
Cre Fw: CACCAGCCAGCTATCAACTCG
Cre Rv: TTACATTGGTCCAGCCACCAG

### 2.2. Gene Expression Analyses

Total RNA was prepared from gastrocnemius muscles or liver using TRIzol (Invitrogen). Complementary DNA was generated from 0.4 μg of RNA reverse-transcribed with SuperScript III Reverse Transcriptase (Invitrogen). Duplicates of cDNA samples were then amplified on the 7900HT Fast Real-Time PCR System (Applied Biosystems, MA, U.S.) using the Power SYBR Green RT-PCR kit (Applied Biosystems). The relative expression ratio of target gene was calculated based on PCR efficiency and quantification cycle deviation (∆Cq) of an unknown sample versus a control, and expressed in comparison to the reference gene [20]. All data were normalized to the reference gene GAPDH expression, of which abundance did not change under any of the experimental conditions, and plotted in arbitrary units as mean ± SEM. The oligonucleotide primers used are shown in Table S1 (Supplementary Materials). FGF21 amplification with cDNA synthesis was obtained from 1.5 to 2 μg of RNA. FGF21 quantification was performed using TaqMan® Universal PCR Master Mix and the specific TaqMan primers FGF21 (Mm 00840165_g1, Life Technologies, MA, USA). Results are expressed as mean ± SEM.

### 2.3. Immunoblotting

Gastrocnemius muscles were lysed and immunoblotted as previously described [21]. Blots were stripped using Restore western blotting stripping buffers (Pierce) according to the manufacturer’s instructions and reprobed when necessary. The membranes were visualized with the ImageQuant LAS 4000 and quantified using ImageJ 1.50 I software (https://imagej.nih.gov/ij/). Protein expression was normalized to Actin or GAPDH. A list of antibodies is indicated in Table S2 (Supplementary Materials). 

### 2.4. Imaging and Transmission Electron Microscopy (EM)

Cryosections of adult gastrocnemius muscles were stained for Hematoxylin & Eosin (H&E) and Succinate dehydrogenase (SDH) staining. Cross-sectional area (CSA) was calculated measuring the cross-sectional area of all individual fibers from entire muscle cross-section from soleus and gastrocnemius muscles based on assembled mosaic image (20× magnification). The morphometric analyses were made using MATLAB Semi-Automatic Muscle Analysis using Segmentation of Histology (SMASH) software. For immunostaining, we used specific antibodies for TOM20 (1:100 dilution in blocking buffer, Santa Cruz) or NCAM (1:200 dilution in blocking buffer, Millipore, St. Louis, MO, USA). WGA conjugated to Cy3 was used to identify the sarcolemma. The images were captured using a Leica DFC300-FX digital charge-coupled device camera and the Leica DC Viewer software. 

For EM, Extensor Digitorum Longus (EDL) muscles were dissected from sacrificed animals, pinned on a Sylgard dish, fixed at room temperature with 3.5% glutaraldehyde in a 0.1 M NaCaCO buffer (pH 7.4), and stored in the fixative solution at 4 °C. Fixed muscles were then post-fixed in a mixture of 2% OsO_4_ and 0.8% K_3_Fe(CN)_6_ for 1–2 h, rinsed with a 0.1 M sodium cacodylate buffer with 75 mM CaCl_2_, en-bloc stained with saturated uranyl acetate, and embedded for EM in epoxy resin (Epon 812) as in [22]. Ultrathin sections (~40 nm) were cut in a Leica Ultracut R microtome (Leica Microsystem, Austria) using a Diatome diamond knife (Diatome Ltd, CH-2501 Biel, Switzerland) and examined at 60 kV after double-staining with uranyl acetate and lead citrate, with an FP 505 Morgagni Series 268D electron microscope (FEI Company, Brno, Czech Republic), equipped with Megaview III digital camera (Munster, Germany) and Soft Imaging System (Germany). 

### 2.5. Mitochondrial Membrane Potential Determination

Mitochondrial membrane potential was measured in isolated fibers from flexor digitorum brevis (FDB) muscles as previously described [5,6]. FDB myofibers were placed in 1 mL Tyrode’s buffer and loaded with 2,5 nM TMRM (Molecular Probes) supplemented with 1 µM cyclosporine H (a P-glycoprotein inhibitor) for 30 min at 37 °C. Myofibers were then observed at an Olympus IMT-2 inverted microscope (Melville, NY) equipped with a CellR imaging system. Sequential images of TMRM fluorescence were acquired every 60 s with a 20X magnification 0.5 numerical aperture UPLANSL objective (Olympus). At the times indicated by arrows, oligomycin (Olm, 5 µM) (Sigma-Aldrich, St. Louis, MO, USA) or the protonophore carbonyl cyanide p-trifluoromethoxyphenylhydrazone (FCCP, 4 µM) (Sigma-Aldrich, St. Louis, MO, USA) were added to the cell culture medium. 

ATP synthase can reversely transport protons across the inner mitochondrial membrane. Due to this fact, the potential is maintained, and in the dysfunctional mitochondria to unmask this issue, we used oligomycin to inhibit ATP-synthase. For this reason, only this treatment allowed us to detect real dysfunctional mitochondria that would inevitably dissipate the potential losing the TMRM signal. Images of TMRM fluorescence were acquired, stored, and analyzed over mitochondrial regions of interest using ImageJ software (http://rsb.info.nih.gov/ij/).

### 2.6. In Vivo FDB Electroporation

Electroporation experiments were performed on FDB muscles from wildtype and knockout animals. The animals were anesthetized by an intraperitoneal injection of xylazine (Xilor) (20 mg/kg) and Zoletil (10 mg/kg). Seven microliters of Hyaluronidase (2 mg/mL) (Sigma-Aldrich, St. Louis, MO, USA) were injected in the feet of anesthetized mice to soften muscle tissue underneath the epidermis. After 50 min, we injected 10 μg of plasmid DNA (mitochondria-targeted mKeima (mt-mKeima)), and after 10 min, electric pulses were applied by two stainless needles placed at 1 cm from each other (100 Volts/cm, 20 pulses, 1 s intervals). Muscles were analyzed 10 days later. No evidence of necrosis or inflammation was observed after the transfection procedure

### 2.7. Mt-mKeima Mitophagy Assay

Mt-mKeima was used to monitor mitophagy in transfected FDB single fibers. Mt-mKeima is a coral-derived protein that exhibits both pH-dependent excitation and resistance to lysosomal proteases. These properties allow for rapid determinations as to whether the protein is in mitochondria or in the lysosome [23]. In fluorescence microscopy, ionized Keima is detected as a red fluorescent signal at lower pH values (lysosome) and neutral Keima is detected as a green fluorescent signal at higher pH values (mitochondria). Fluorescence of mt-mKeima was imaged in two channels via two sequential excitations (458 nm, green; 561 nm, red) and using a 570-to-695 nm emission range. The level of mitophagy was defined as the total number of red pixels divided by the total number of all pixels.

### 2.8. Force Measurements

In vivo gastrocnemius force measurements were performed as described previously [24]. Mice were anesthetized and stainless steel-wired electrodes were placed on each side of the sciatic nerve. Torque production of the plantar flexors was measured using a muscle lever system (Model 305c; Aurora Scientific, Aurora ON, Canada). The force–frequency curves were determined by increasing the stimulation frequency in a step-wise manner, pausing for 30 s between stimuli to avoid effects due to fatigue. Following force measurements, animals were sacrificed by cervical dislocation and muscles were dissected and weighed. Force was normalized to the muscle mass as an estimate of specific force.

### 2.9. Autophagic Flux Quantification

We monitored autophagic flux in basal conditions using colchicine [6,23,25]. Briefly, inducible transgenic mice were treated with 0.4 mg/kg of colchicine or vehicle by intraperitoneal injection. The treatment was repeated twice every 12 h prior to muscle harvesting.

### 2.10. Protein Carbonyls Detection

Carbonylation of proteins from soleus muscles were detected by using the OxyBlot Protein Oxidation Detection Kit (Millipore St. Louis, MO, USA), s7150) [6]. The quantification analysis was performed with ImageJ Software and all values were normalized for the housekeeping GAPDH. 

### 2.11. Blood Glucose Measurement

The mice fasted for 8 h before analysis during their normal rest/fasting phase. Blood glucose was measured using a Glucocard G+ meter (Arkray Factory, Shiga, Japan).

### 2.12. Plasma FGF21 Measurement

The serum was obtained from the blood collected from controls and DKO mice. Blood FGF21 levels were determined using a Rat/Mouse FGF21 Enzyme-linked Immunosorbent ELISA-Kit (Merck Millipore, St. Louis, MO, USA, EZRMFGF21-26K), adhering to the manufacturer’s instructions. All data are expressed in pg/mL.

### 2.13. Statistical Analysis

All data are expressed as means ± SEM of independent experiments. Statistical analysis was performed using two-tailed Student’s *t*-test or 2-way analysis of variant (ANOVA). When ANOVA revealed a significant difference, further analysis was performed using Bonferroni’s multiple comparison test. The statistical significance threshold was set at *p* < 0.05. * compared to control, # compared to DKO 70 days, $ compared to DKO 90 days, & compared to DKO 120 days, and £ versus DKO 150 days.

## 3. Results

### 3.1. Acute Simultaneous Deletion of Opa1 and Drp1 in Adult Muscles Induces Muscle Atrophy and Weakness

**Figure 1:**
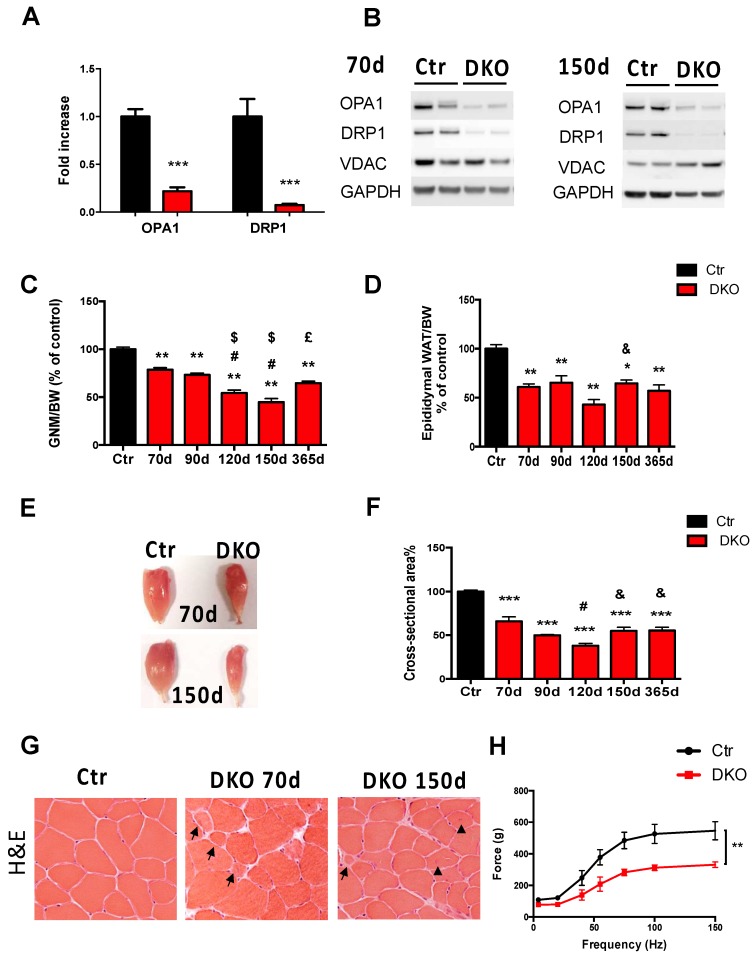
Losses of Opa1 and Drp1 in adult mice cause body weight (BW) loss, muscle atrophy, and muscle weakness. Opa1 and Drp1 mRNA (**A**) and protein levels (**B**) were decreased in DKO gastrocnemius (GNM) muscles. (**C**) Wet weight of GNM muscle normalized to BW and relative to control. (**D**) Epididymal fat content normalized to BW was decreased in DKO mice. (**E**) Dissected GNM muscles from control and DKO mice showed an important amount of atrophy after 70 and 150 days from tamoxifen treatment. (**F**) The quantification of cross-sectional areas within GNM individual myofibers confirms muscle atrophy in DKO mice. (**G**) Representative Hematoxylin & Eosin (H&E) staining of GNM. The arrows indicate very small fibers in both images “DKO 70d” and “DKO 150d”. Arrowheads in the image “DKO 150d” show the central nuclei. (**H**) Force measurements performed in vivo on GNM muscles. Absence of Opa1 and Drp1 leads to a significant decrease in absolute force. Data are shown as means ± SEM. Two-tailed unpaired Student’s *t*-test and 2-way analysis of variant (ANOVA) were used. Statistical significance: * *p* < 0.05, ** *p* < 0.01**, *** *p* < 0.001, compared to control; # compared to DKO 70d; $ compared to DKO 90d; & compared to DKO 120d; and £ versus DKO 150 days; 70 days, 90 days, 120 days, 150 days, and 365 days refer to days from the beginning of tamoxifen treatment.

Muscle-specific Opa1 knockout mice display fragmented mitochondria, muscle loss, and weakness which reverberates to the whole body and leads to premature aging and then mortality [6]. To explore whether inhibition of fusion might be compensated by a concomitant blockage of fission, we concomitantly deleted OPA1 and the critical fission protein DRP1. Thus, we generated muscle-specific tamoxifen-inducible Opa1/Drp1 DKO mice. Tamoxifen treatment started in 5-month-old adult mice, and mice were sacrificed to collect muscles at different points of time between 70 and 365 days (Figure S1A, Supplementary Materials). OPA1 and DRP1 transcripts (Figure 1A) and proteins (Figure 1B; Figure S1B, Supplementary Materials) were efficiently reduced at both 70 and 150 days. Interestingly, within 35 days from the beginning of tamoxifen treatment, both male and female DKO mice began losing body weight (Figure S1C,D, Supplementary Materials). The reduction of body weight is secondary to a significant decrease in muscle mass (Figure 1C) and white adipose tissue (WAT) (Figure 1D). There was a progressive reduction of DKO gastrocnemius muscle mass (Figure 1E), and the cross-sectional areas of individual fibers were significantly reduced by 35%, 50%, 60%, and 45% at 70, 90, 120, and 150 days, respectively, compared to that of controls (Figure 1F). Interestingly, muscle loss stabilized from 150 to 365 days after the deletion (Figure 1F). Importantly, muscle loss occurred not only in gastrocnemius muscles but also in soleus, a muscle with different metabolic properties, and fiber type composition (slow oxidative fibers) (Figure S1E, Supplementary Materials). Muscle histology analyses by H&E staining of transverse muscle sections revealed the presence of very small fibers with no signs of inflammation, degeneration, or regeneration at 70 days (Figure 1G). However, extending Opa1 and Drp1 ablation in muscles for 120 days, 150 days, and 365 days leads to myofiber death and the appearance of center-nucleated fibers in approximately 6%–8% of gastrocnemius fibers (Figure 1G; Figure S1F, Supplementary Materials). Importantly, mitochondria-rich oxidative fibers of soleus muscles display a more severe myopathic phenotype, when compared to gastrocnemius, as revealed by the dramatic increase of center-nucleated fibers (33%) (Figure S1G, Supplementary Materials). To investigate if muscle loss correlates with muscle weakness, we measured the force generated by gastrocnemius muscles in living animals at 70 days after tamoxifen treatment. Maximal absolute force (tetanic force) was significantly reduced (Figure S1H, Supplementary Materials), suggesting an increased loss of sarcomeric proteins. Moreover, we observed a significant decrease in DKO mice when we analyzed the force normalized for muscle mass. These results indicated that, besides a loss in mass, the skeletal muscles of DKO mice also had a lower intrinsic muscle force, revealing a myopathic phenotype (Figure 1H). Thus, blocking mitochondrial dynamics in adult muscles causes WAT loss, progressive atrophy, and weakness.

### 3.2. Dampening Mitochondrial Dynamics Leads to Alterations in Mitochondrial Morphology, Distribution, and Function

**Figure 2:**
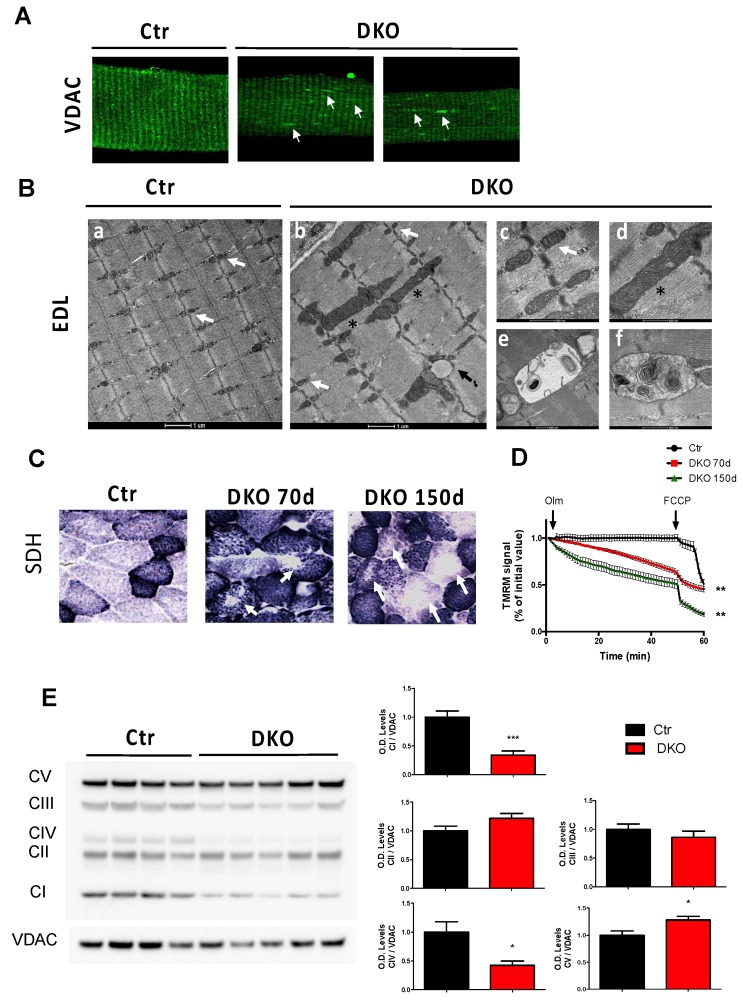
Double-knockout (DKO) muscles have alterations in mitochondrial morphology and function. (**A**) Confocal microscopy of single isolated flexor digitorum brevis (FDB) fibers immunostained for Voltage-Dependent Anion Channel (VDAC). Arrows show longitudinally elongated mitochondrial structures in DKO muscles. (**B**) Representative electron micrographs of EDL muscles of controls and DKO mice. Control mitochondria (pointed by white arrows in insets a and c) were placed in proximity of Z lines and usually exhibited an electron-dense matrix. DKO muscles displayed a mixture population of normal mitochondria (indicated by white arrows in inset b), larger longitudinally oriented mitochondria (indicated by asterisks in insets b and d), and mitochondria with disrupted (inset e) or onion-like cristae (inset f). (**C**) Succinate dehydrogenase SDH staining of GNM sections indicating the presence of bigger mitochondria in some fibers from 70 days and 150 days DKO mice. White arrows indicate central fiber areas depleted of mitochondria. (**D**) Absence of Drp1 and Opa1 induced mitochondrial depolarization in FDB muscle fibers from DKO 70 days and 150 days mice. Isolated adult FDB fibers were loaded with TMRM. Oligomycin (Olm) and FCCP were added at the indicated time points. (**E**) Immunoblot and densitometric analysis of mitochondria OXPHOS complexes levels. complex I (CI) and complex IV (CIV) were significantly decreased in GNM muscles from adult DKO mice. Data are shown as mean ± SEM. Two-tailed unpaired Student’s *t*-test was used. Statistical significance: * *p* < 0.05, ** *p* < 0.01**, *** *p* < 0.001, compared to control.

The maintenance of a functional mitochondrial network, by continuously alternating fusion and fission, is crucial for healthy muscle. [3]. Thus, we investigated the impact on mitochondrial morphology and function of the combined abrogations of Opa1 and Drp1. Confocal microscopy on 70 days DKO mice isolated fibers that were immunostained for the mitochondrial protein Voltage-Dependent Anion Channel (VDAC) reported the typical mitochondrial network striation as seen in controls (Figure 2A). However, we noticed the presence of longitudinal VDAC-positive elongated structures in DKO fibers (Figure 2A, indicated by white arrows). Ultrastructural analysis by electron microscopy of 70 days DKO EDL muscles revealed a regular sarcomeric structure with the accumulation of lipid droplets (Figure 2B, inset b, indicated by the black arrow). Mitochondria were heterogeneous in size and morphology. Sixty-five percent of the observed mitochondria were similar to the controls with an electron-dense matrix, parallel internal cristae and placed at I band in the proximity of Z lines (Figure 2B, insets b and c, indicated by white arrows). However, 25% of mitochondria were larger elongated mitochondria, longitudinally oriented between myofibrils (Figure 2B, insets b and d, indicated by asterisks), and 10% were mitochondria with disrupted cristae structures (Figure 2B, inset e) or with onion-like inner membrane structures (Figure 2B, inset f). SDH staining showed bigger puncta in muscle fibers, suggesting that mitochondria are larger in DKO mice than in control muscles (Figure 2C). Interestingly, several fibers displayed central areas depleted of mitochondria (Figure 2C, indicated by white arrows) in both 70 days and 150 days DKO muscles. This resembled a central core disease where regions of myofiber cytoplasm were completely devoid of mitochondria. Mitochondria from DKO FDB myofibers underwent a significant depolarization when F1F0-ATPase was inhibited by oligomycin, suggesting that these fibers were in part relying on the reverse activity of ATP synthase to preserve their membrane potential because of proton leak-consuming proton motive force, in line with the morphological abnormalities. Of note, mitochondrial depolarization was enhanced in 150 days DKO mice compared to in 70 days DKO mice (Figure 2D). Western blot analyses of the subunits of the different respiratory chain complexes revealed a reduction of complexes I and IV and a slight increase of complex V in DKO mice compared to that in controls (Figure 2E). Thus, the disruption of both mitochondrial fission and fusion in the adult skeletal muscles leads to alterations in mitochondrial morphology, distribution, and function. 

### 3.3. Combined Acute Ablation of Mitochondrial Fusion and Fission in Adult Muscles Leads to ER Stress, Activation of Ubiquitin–Proteasome System, and the Inhibition of both Bulk Autophagy and Mitophagy

**Figure 3:**
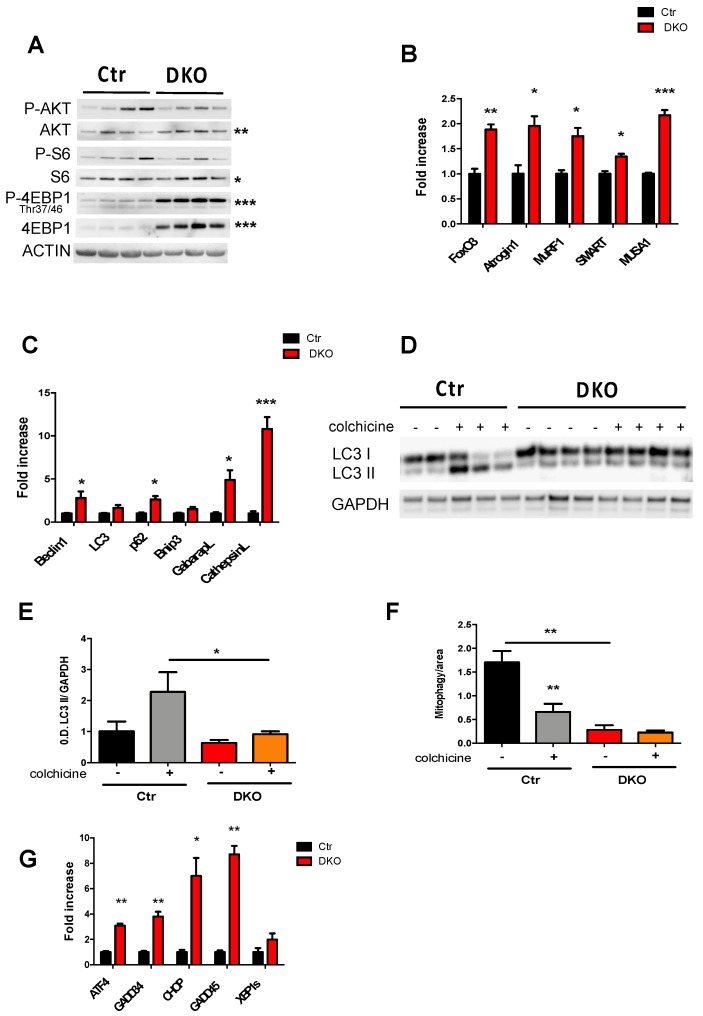
Protein degradation pathways were activated with acute inhibition of Opa1 and Drp1 in adult muscles. (**A**) Total protein extracts from GNM muscles were immunoblotted with the indicated antibodies. Statistical significance of densitometric analysis is indicated on the right. Data are normalized to actin. (**B**) RT-PCR analysis of FoxO-dependent transcripts showing increase in GNM DKO muscles of FoxO3, and the E3 ubiquitin ligases, Atrogin1, MuRF1, SMART and MUSA1. (**C**) Quantitative PCR analysis of autophagy-related transcripts showed a significant induction of autophagy markers in GNM DKO muscles. Immunoblot (**D**) and relative densitometric quantification (**E**) of GNM muscle lysates from colchicine treated mice showed a block in autophagy flux after Opa1 and Drp1 inhibition. (**F**) Mitophagy flux was analyzed by electroporation of a reporter plasmid (mitochondria-targeted mKeima (mt-mKeima)) into flexor digitorum brevis muscles of adult control and DKO mice, and changes of fluorescent spectra were detected and normalized to the fiber area. (**G**) RT-PCR analysis showing the activation of PERK-dependent Unfolded Protein Response in GNM muscles. Data are shown as mean ± SEM. Two-tailed unpaired Student’s *t*-test and 2-way ANOVA were used. Statistical significance: * *p* < 0.05, ** *p* < 0.01, *** *p* < 0.001, compared to control.

Alterations in mitochondrial morphology and function have severe consequences for the maintenance of muscle mass and function [3,5,6]. The regulation of muscle size depends on the balance between protein synthesis and protein degradation. Thus, we first investigated the well-established IGF1-AKT-mTOR pathway, which is known to contribute to the regulation of protein synthesis in skeletal muscle (Figure 3A). In fact, activation of IGF1-AKT-mTOR pathway induces hypertrophy while its inhibition leads to muscle atrophy [21]. While AKT phosphorylation was not affected, the total AKT increased in 70 days DKO muscles. The mTOR downstream targets S6 S240/244 and 4EBP1 Thr37/46 were activated differently in DKO muscles. Phospho-4EBP1 Thr37/46 and total 4EBP1 increased, while there were no differences in the phosphorylation levels of S6 S240/244 despite the increase in total S6 (Figure 3A; Figure S2A, Supplementary Materials). Thus, the AKT-mTOR pathway is not consistent and does not explain the observed muscle atrophy. Then, we checked the catabolic pathways, of which activation relies on gene expression programs. FoxOs transcription factors are critical mediators of the catabolic response during atrophy, controlling half of the atrophy-related genes including the UPS and ALS systems [25]. FoxO3, as well as its downstream target genes, the E3 ligases, Atrogin 1, MuRF1, SMART and MUSA1, were induced in 70 days DKO muscles (Figure 3B). In line with muscle mass stability seen 150 days after tamoxifen treatment (Figure 1F), FoxO3, Atrogin 1, and MuRF1 progressively decreased from 150 to 365 days (Figure S2B, Supplementary Materials). We then checked the transcriptional levels of the FoxO-dependent autophagy-related genes and we found an upregulation of most of these genes in DKO mice (Figure 3C). Thus, the next step was to monitor the autophagic flux, in vivo, by using colchicine, to disrupt the autophagosome delivery to lysosome (Figure 3D). As expected, LC3 II accumulated in control muscles upon colchicine treatment, indicating that autophagy was ongoing in these muscles (Figure 3D,E). In contrast, LC3 II accumulation was significantly reduced in DKO colchicine-treated muscles (Figure 3E). These findings indicated an impairment of general autophagy flux. Next, we checked the autophagy-dependent selective degradation of the mitochondria, named mitophagy. Adult muscles were transfected with mt-mKeima, a pH-sensitive mitochondrial targeted probe that changes the fluorescence upon acidification. Therefore, this probe reflects the autophagy-dependent delivery of mitochondrial to lysosomes. The measurements showed that mitophagy was blocked in DKO muscles (Figure 3F). Finally, since mitochondrial dysfunction and ER stress are strictly associated, they are both a consequence of OPA1 inhibition. We analyzed the transcriptional levels of ATF4 that transcribes several genes including *GADD34*, *CHOP*, and *GADD45* during chronic ER stress. All these genes were upregulated in 70 days DKO muscles which indicated the activation of a PERK-dependent Unfolded Protein Response (UPR) (Figure 3G). Importantly, the overexpression of ATF4 and GADD45 are sufficient to induce muscle loss [26,27]. In conclusion, the signaling pathways impinging on muscle loss due to the combined abrogation of mitochondrial fusion and fission involved the activation of the UPS and the impairment of general autophagy, as well as mitophagy flux and the activation of UPR.

### 3.4. Blocking Mitochondrial Fission in Muscle-specific OPA1-Null Mice Mitigates Aging Sarcopenia, Blunts Oxidative Stress, and Rescues Lethality. 

**Figure 4:**
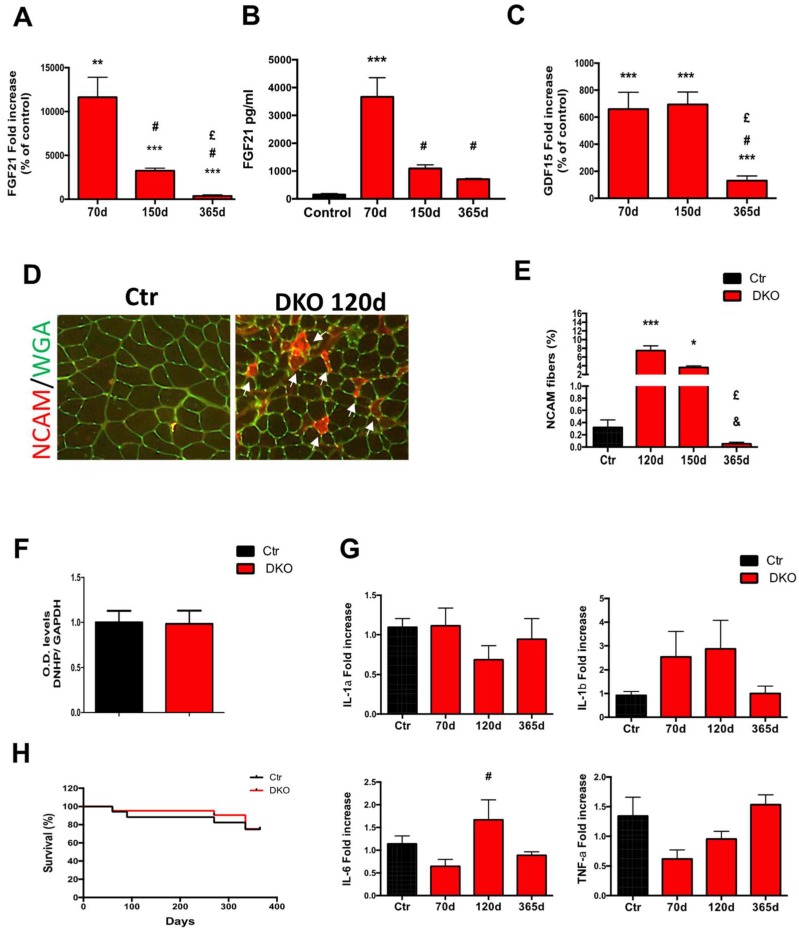
Inhibition of Drp1 in muscle-specific Opa1 knockout mice alleviates FGF21 induction, denervation, oxidative stress, inflammation and prevents mortality: (**A**) FGF21 mRNA GNM muscle levels and serum levels (**B**) of control and DKO mice; (**C**) GDF15 mRNA levels of control and DKO GNM muscles; (**D**) representative image showing NCAM-positive fibers in GNM DKO muscles (white arrows); (**E**) quantification of denervated NCAM-positive fibers of GNM muscles; (**F**) densitometric quantification of the carbonylated proteins in control and DKO soleus muscle extracts; (**G**) mRNA levels of the inflammatory cytokines IL-1α, Interleukin 1-beta (IL-1β), IL-6, and Tumour Necrosis Factor-alpha (TNFα) in GNM muscles. (**H**) Kaplan–Meier survival curve of DKO mice from the beginning of tamoxifen treatment (day zero). Data are shown as mean ± SEM. Two-tailed unpaired Student’s *t*-test and 2-way ANOVA were used. Statistical significance: * *p* < 0.05, ** *p* < 0.01, *** *p* < 0.001, compared to control; # compared to DKO 70d; & compared to DKO 120d; and £ versus DKO 150d.

Acute inhibition of Opa1 in skeletal muscles led to the premature death of 8-month-old adult mice within 120 days from the tamoxifen-induced deletion [6]. Moreover, these mice presented a phenotype of sarcopenia and premature and unhealthy aging, in just a few weeks they recapitulated 24–30 months of life. We showed that Opa1-null muscles released FGF21 to mediate an integrated stress response which causes oxidative stress, systemic inflammation, metabolic alterations, precocious senescence of different epithelial tissues, and premature death [6]. Since mitochondrial dysfunction triggers the expression in muscle of FGF21 and GDF15, which positively correlate with unhealthy aging and mortality [6,28], we measured the transcript levels of these hormones in DKO muscles. The levels of Fgf21 in muscle (Figure 4A) and blood (Figure 4B) peaked in 70 days DKO but then were progressively reduced and then reached their control values at 365 days. GDF15 levels dramatically increased at 70 days and 150 days DKO muscles (Figure 4C). However, after one year from the OP1/DRP1 deletion, GDF15 levels were almost reduced to control levels (Figure 4C). Adult DKO mice showed an evident kyphosis at 90 days (Figure S3A, Supplementary Materials), which was partially reverted at 150 and 365 days after the deletion, keeping in line with FGF21 and GDF15 levels. These data match with muscle loss, which was partially recovered at 150 days and 365 days DKO mice (Figure 1F). Suggesting a problem of myofiber innervation, the NCAM marker of denervation started to be expressed at 120 days in 8% of DKO fibers, but then it reverted to the control values at 365 days (Figure 4D,E). Interestingly, at 150 d, the percentage of NCAM-positive fibers was similar in both gastrocnemius (Figure 4E) and soleus muscles (Figure S3B, Supplementary Materials). Additionally, several denervation markers like Musk1, AchR gamma, Runx1, and myogenin peaked at 120 days and 150 days, and returned to control levels after 365 days from tamoxifen treatment (Figure S3C, Supplementary Materials). Like in Opa1-deleted mice, DKO mice had several metabolic alterations at 70 days such as reduced WAT (Figure 1D), liver steatosis (Figure S3D, Supplementary Materials) and low serum glucose levels (Figure S3E, Supplementary Materials). Consistent with the reduction of muscle and serum FGF21 in 150 days and 365 days DKO mice (Figure 4A,B), WAT was partially recovered (Figure 1D), liver steatosis ameliorated already at 120 days (Figure S3D), and glycemia was restored to normal levels at 150 days after tamoxifen treatment (Figure S3E, Supplementary Materials). Muscle-specific deletion of Opa1 triggered oxidative stress and upregulation of Interleukin 6 (IL-6) and Interleukin 1-alpha (IL-1α) in muscles [6]. The combined genetic ablation of fusion and fission genes completely prevented oxidative stress (Figure 4F; Figure S3F, Supplementary Materials) and slightly increased only IL-6 expression in DKO muscles at 120 days (Figure 4G). Finally, the survival curve of DKO mice was superimposed with control mice (Figure 4H). Thus, the simultaneous deletion of Opa1 and Drp1 prevented the precocious death of Opa1 knockout mice, counteracted oxidative stress, muscle and WAT loss, and prevented an inflammatory response. In conclusion, inhibition of mitochondrial fission in Opa1-null muscles mitigates FGF21 induction, denervation, oxidative stress, and inflammation, rescuing the lethal phenotype of Opa1 knockout mice. 

## 4. Discussion

Here, we identified that the simultaneous reduction of Opa1 and Drp1 in skeletal muscle acts as a compensatory mechanism to alleviate the detrimental effects of unbalanced mitochondrial dynamics (Table S4, Supplementary Materials). Mitochondrial fusion and fission play crucial roles in the homeostatic control of muscle mass [3]. Accordingly, the failure in mitochondrial-shaping processes has detrimental consequences for the maintenance of muscle mass and function [4,5,6,7,8,13,29]. The experimental models studied, thus far, have individually explored the impact in skeletal muscle of the imbalanced fission or fusion processes [4,5,6,7,8,13,29]. However, muscle loss in aging sarcopenia, cancer cachexia, and myasthenia gravis are characterized by the decline of both fusion machinery and fission machinery [6,11,12,15,16,17,18]. 

To address the physiological relevance of the concomitant abrogation of mitochondrial fusion and fission events in skeletal muscle, we generated muscle-specific Opa1 and Drp1 DKO mice. First, we attempted to generate a conditional DKO model by crossing OPA1 and DRP1-floxed mice with Myosin Light Chain-Cre transgenic mice to specifically delete OPA1 and DRP1 in skeletal muscle during embryogenesis [30]. However, analysis of the genotype of the expected Mendelian distribution of more than 100 pups identified no homozygous mice with the double deletion of OPA1 and DRP1, suggesting that homozygous DKO mice died in utero (Table S3, Supplementary Materials). This finding underscores the importance of the dynamic control of mitochondrial shape, by fusion and fission, to maintain a healthy mitochondrial network during muscle development. The impossibility of obtaining viable mice hindered further analysis. We, therefore, generated tamoxifen-inducible muscle-specific Opa1/Drp1 DKO mice. 

The abolishment of mitochondrial dynamics in adult muscles resulted in muscle loss and weakness. Mechanistically, muscle loss is due to FoxO-mediated activation of the ubiquitin–proteasome system together with the impairment of general autophagy and mitophagy pathways [25,31,32]. While unbalanced mitochondrial fusion and fission lead to elongated and fragmented mitochondria, respectively [3], it is not known if the concomitant loss of both processes can rescue mitochondria shape in skeletal muscle. Electron microscopy analyses revealed the presence of a heterogeneous mitochondrial population in DKO muscles. More than 65% of the observed mitochondria resembled control mitochondria, 25% were abnormally enlarged, and 10% displayed abnormal inner organization. Similar changes were observed in a recent study that investigated the acute downregulation of both Opa1 and Drp1 in *Drosophila* adult muscles [33]. A possible explanation for the observed ultrastructural mitochondria alterations is an unbalanced Mfn1-dependent outer mitochondrial membrane fusion in DKO muscles, while the fusion of the inner membrane together with mitochondrial fission is absent. The impossibility to maintain a healthy mitochondrial network by the continuous reshaping of the organelle in DKO muscle leads to dysfunctional mitochondria which accumulate due to a block in mitophagy. Importantly, the resulting mitochondrial dysfunction leads to the activation of ER stress, UPR, and FGF21 pathways which further contribute to muscle atrophy. In fact, the overexpression of either Atf4 [27], Gadd45 [26], or Fgf21 [23] in muscles is sufficient to induce muscle atrophy. The initial increase of Fgf21 in 70 days DKO muscles and blood can explain the observed metabolic changes such as basal hypoglycemia and reduced epididymal pads. Since we have recently established the contribution of unopposed mitochondrial fission to aging sarcopenia and lethality [6], we addressed whether balancing the loss of mitochondrial fusion by inhibiting fission-reverted Opa1 phenotype. Here, we showed that muscle-specific DKO mice had a less severe phenotype than Opa1 knockout mice which were dependent on muscle Gdf15 and Fgf21 levels. In Opa1-deleted mice, chronic elevation of muscle-derived circulating Fgf21 mediated important metabolic changes, systemic inflammatory response, precocious senescence of epithelial tissues, and premature animal death [6]. Moreover, in humans, Fgf21 levels are inversely correlated with survival [28]. Conversely, other reports have associated high levels of circulating Fgf21 with the extension of lifespan [34]. The discrepancy in the two observations can most likely be explained by the threshold effect of Fgf21, where moderate levels result in adaptive responses to stress, while exacerbated levels are detrimental to stress [28]. We can speculate that the initial exacerbated increase in 70 days DKO muscles is important to activate stress responses, while the reduction of Fgf21, which started at 150 days, might be an attempt to maintain Fgf21 levels within the threshold/range to guarantee a hormetic response by triggering compensatory mechanism that mitigates Fgf21 effects. It is important to note that muscle loss increased progressively within 120 days from the start of tamoxifen treatment. However, from 150 to 365 days after the deletion of Opa1 and Drp1, the transcriptional atrophy program was blunted, resulting in a partial muscle mass recovery which correlates to reduced Fgf21 muscle levels [23], despite persistent mitochondrial dysfunction in DKO muscles. Consistent to reduced Fgf21 induction in 150 days DKO muscles, glycemia was normalized, and WAT was partially spared after 150 days compared to that in 120 days DKO muscles when muscle atrophy is maximal. Despite a dramatic reduction in Fgf21 in 365 days DKO muscles, lipolysis might not be completely blocked because UCP1 is induced in WAT [6]. Moreover, muscle denervation, oxidative stress, and inflammation are mitigated in DKO muscles, rescuing the lethal phenotype of Opa1 knockout mice. Like skeletal muscle, the specific abrogation of mitochondrial dynamics in the heart leads to less severe cardiomyopathy and reverts mortality in respect to unopposed fission or fusion heart-specific mice models [35]. Overall, our results suggested that DKO is a genetic model that resembles a phenotype of aging sarcopenia (Table S5, Supplementary Materials). Muscles display muscle loss, autophagy/mitophagy flux impairment, and a mixed population of both enlarged and normal mitochondria, as well as mild mitochondrial dysfunction in the absence of oxidative stress.

Interestingly, mild mitochondrial stress might elicit beneficial effects on health and lifespan through the activation of mitochondrial hormesis [28,36]. Thus, the concomitant decline of mitochondrial dynamics in aging sarcopenia might be necessary to activate a mild mitochondrial dysfunction and thus mitohormesis, to promote healthy aging while unhealthy/accelerated aging associates to unopposed mitochondrial fission. 

## Supplementary Materials

The following are available online at https://www.mdpi.com/2073-4409/8/6/597/s1 [37,38,39,40,41], Figure S1, Figure S2, Figure S3, Table S1: List of primers used for quantitative PCR analysis, Table S2: Antibodies used in this study Table S3: Genotyping distribution of MLC-DKO mice, Table S4: Comparison between inducible muscle-specific Opa1, Drp1 and DKO phenotype and signaling, and Table S5: Comparison between DKO muscles and sarcopenic muscles.
