# Supplementary material for: Inhibition of the Fission Machinery Mitigates OPA1 Impairment in Adult Skeletal Muscles
Source: Cells. 2019 Jun 15;8(6):597. doi: 10.3390/cells8060597 (PMC6627087; doi:10.3390/cells8060597)
Supplement: Supplementary file 1 [file cells-08-00597-s001.pdf]

FIGURE S1

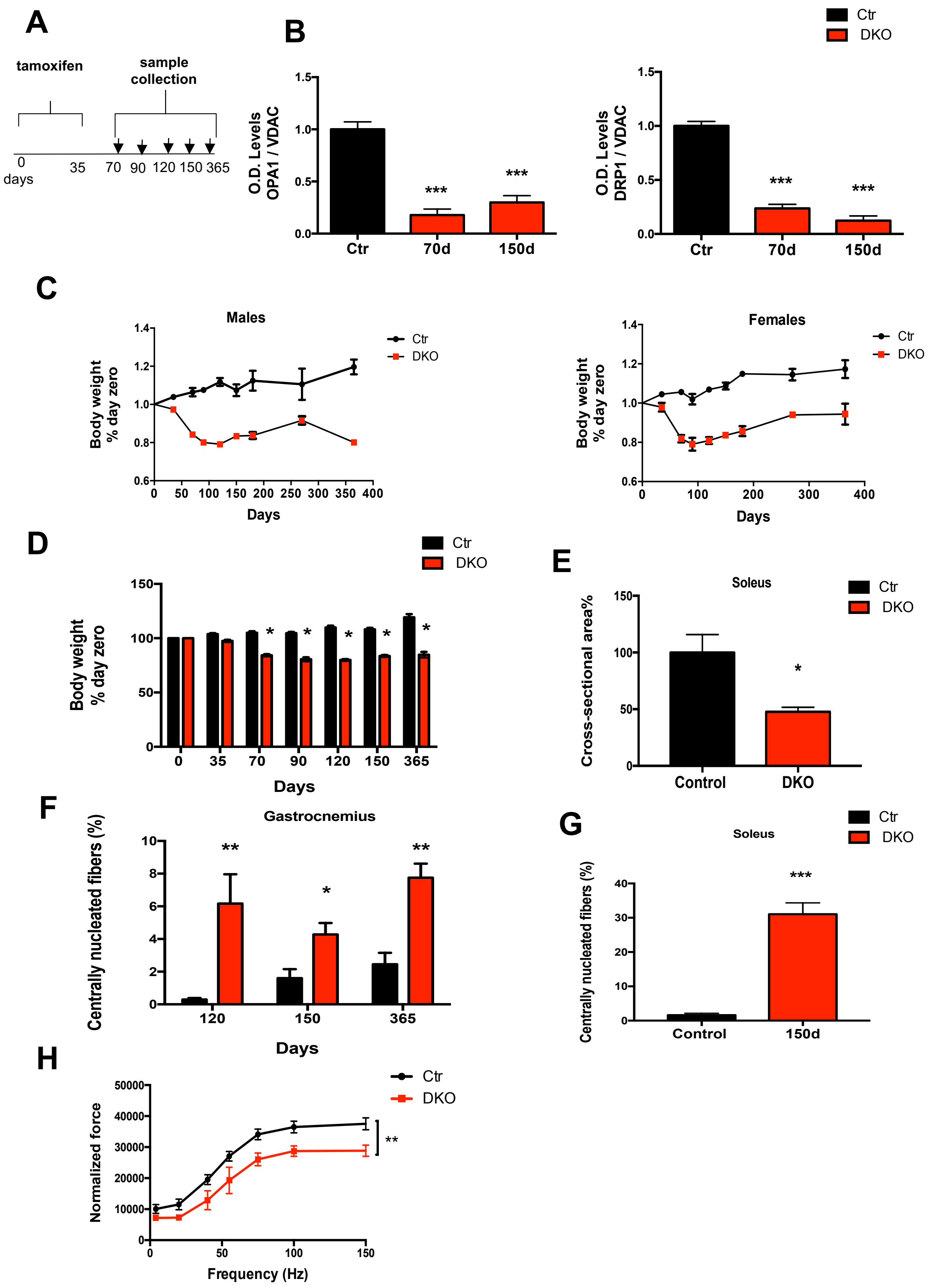

A
tamoxifen
sample collection
0 35 70 90 120 150 365
days
B
O.D. Levels OPA1 / VDAC
O.D. Levels DRP1 / VDAC
Ctr
70d
150d
***
Ctr
DKO
C
Males
Females
Body weight % day zero
Days
Ctr
DKO
D
Body weight % day zero
Days
0 35 70 90 120 150 365
Ctr
DKO
E
Soleus
Cross-sectional area%
Control
DKO
Ctr
DKO
F
Gastrocnemius
Centrally nucleated fibers (%)
120 150 365
Days
G
Soleus
Centrally nucleated fibers (%)
Control
150d
Ctr
DKO
H
Normalized force
Frequency (Hz)
Ctr
DKO
**

Figure S1: A) Scheme showing tamoxifen treatment and time points of samples collection. Samples were collected at the time points indicated with arrows. **B)** Densitometric analysis of Opa1 and Drp1 normalized to VDAC. **C)** Body weight of both, males (left) and females (right) versus days from the beginning of tamoxifen treatment (day zero). **D)** Body weight of control and DKO littermates at different time points normalized to the first day of tamoxifen treatment (day zero). **E)** Quantification of cross-sectional area of soleus individual myofibers confirms muscle atrophy in DKO mice. **F)** Centrally nucleated fiber in gastrocnemius muscles at 120, 150 and 365 days after tamoxifen treatment and in soleus muscles **(G) H)** Force measurements performed in vivo on gastrocnemius muscles. Absence of Opa1 and Drp1 leads to a significant decrease in specific muscle force, normalized to gastrocnemius wet weight. Data are shown as mean ± s.e.m. Two-tailed unpaired Student's t test and 2-way analysis of variant (ANOVA) were used. Statistical significance p< 0.05*, p< 0.01**, p< 0.001*** compared to control, # compared to DKO 70d, $ compared to DKO 90d, & compared to DKO 120d, and £ versus DKO 150d.

70d, 90d, 120d, 150d, and 365d refers to days from the beginning of tamoxifen treatment.

FIGURE S2

A

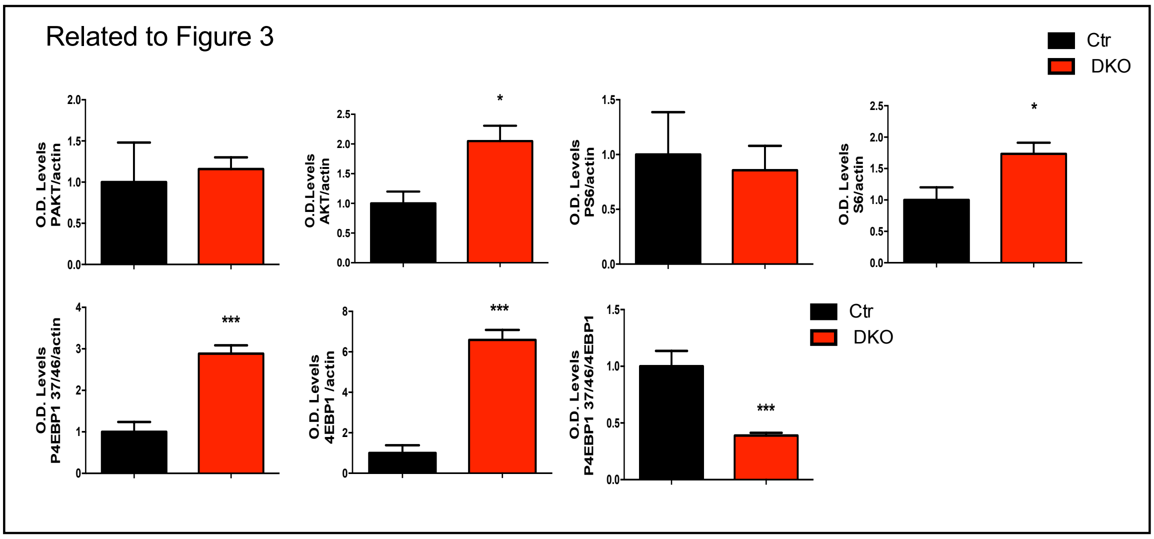

B

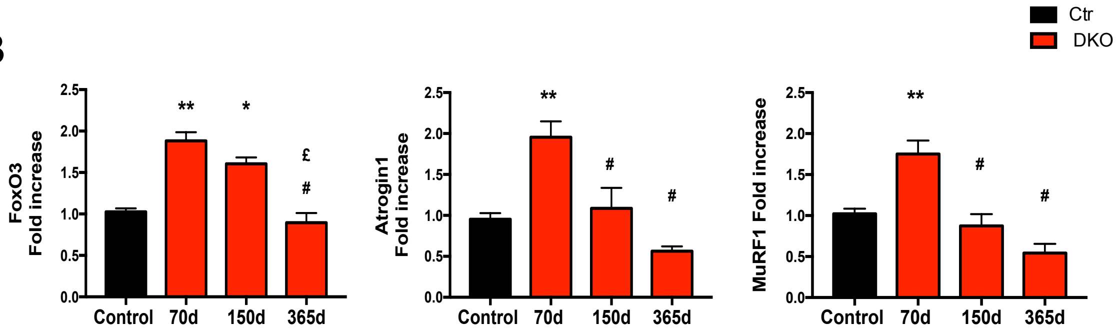

Figure S2: A) Densitometric analysis of the western blots related to Figure 3. B) mRNA level of FoxO3, Atrogin1 and MuRF1 in gastrocnemius muscles at different time points.

FIGURE S3

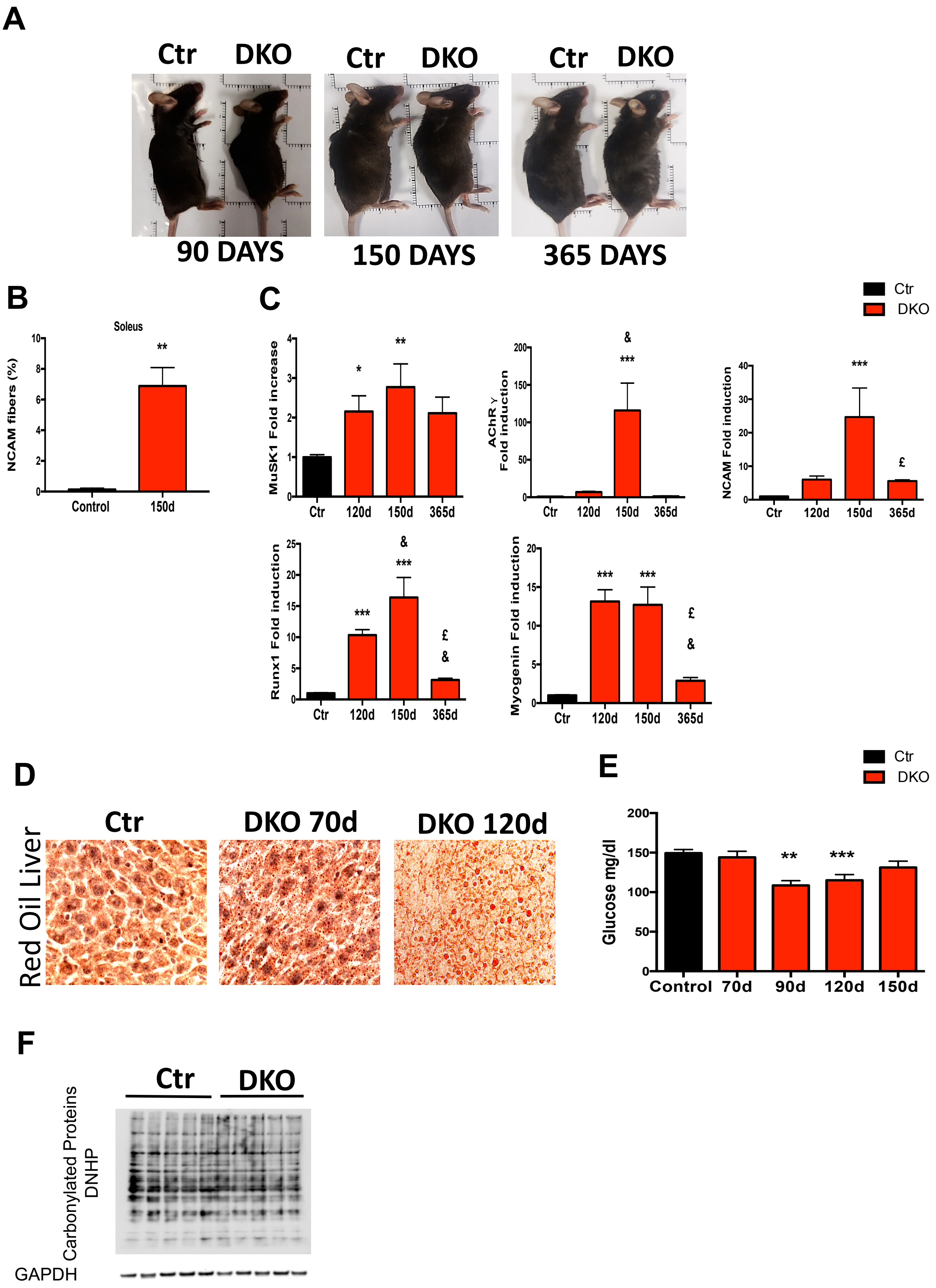
A
Ctr
DKO
Ctr
DKO
Ctr
DKO
90 DAYS
150 DAYS
365 DAYS
B
Soleus
NCAM fibers (%)
**
Control
150d
C
Ctr
DKO
MuSK1 Fold increase
*
**
AChR γ Fold induction
&
***
NCAM Fold induction
***
£
Ctr
120d
150d
365d
Runx1 Fold induction
***
&
***
£
&
Myogenin Fold induction
***
***
£
&
D
Red Oil Liver
Ctr
DKO 70d
DKO 120d
E
Ctr
DKO
Glucose mg/dl
**
***
Control
70d
90d
120d
150d
F
Ctr
DKO
Carbonylated Proteins
DNHP
GAPDH

Figure S3: A) Kyphosis of adult DKO mice is evident 90 days after the deletion of Opa1 and while it is partially reverted at 150d and 365 days. **B)** Quantification of denervated NCAM-positive fibers of soleus muscles. **C)** mRNA levels of the denervation markers MuSK1, AchRγ, NCAM, Runx1 and Myogenin. **D)** Representative images of oil red O staining in liver cryosections. **E)** Glycemia in control and DKO mice. **F)** Representative oxyblot of control and DKO samples.

TABLE S1

	Forward primer (5'-3')	Reverse primer (3'-5')
**AchRγ**	AGTGCAGGCAGTATTGGAGA	AGGTTACAGGCATCCACACAG
**ATF4**	TCCTGAACAGCGAAGTGTTG	ACCCATGAGGTTTCAAGTGC
**Atrogin1**	GCAAACACTGCCACATTCTCTC	CTTGAGGGGAAAGTGAGACG
**Beclin1**	TGGAAGGGTCTAAGACGT	GGCTGTGGTAAGTAATGGA
**Bnip3**	TTCCACTAGCACCTTCTGATGA	GAACACCGCATTTACAGAACAA
**CathepsinL**	GTGGACTGTTCTCACGCTCAAG	TCCGTCCTTCGCTTCATAGG
**CHOP**	GCTGGAAGCCTGGTATGAG	ATGTGCGTGTGACCTCTGTT
**Drp1**	TCAGATCGTCGTAGTGGGAA	TCTTCTGGTGAAACGTGGAC
**FoxO3**	GTATGGCGTTTGTGAGAACC	AGCCCCAAAATGTGTCTGTA
**GabarapL**	CATCGTGGAGAAGGCTCCTA	ATACAGCTGGCCCATGGTAG
**GADD34**	AGAGAAGACCAAGGGACGTG	CAGCAAGGAATGGACTGTG
**GADD45**	GAAAGTCGCTACATGGATCAGT	AAACTTCAGTGCAATTTGGTTC
**GAPDH**	CACCATCTTCCAGGAGCGAG	CCTTCTCCATGGTGGTGAAGAC
**IL-1a**	TCTCCTTCTCCTCCTTCTCC	GCTCCCTAAGTTCCCTGTCA
**IL-1b**	AAGGAGAACCAAGCAACGACAAAA	TGGGGAACTCTGCAGACTCAAACT
**IL6**	TAGTCCTTCCTACCCCAATT	TTGGTCCTAAGCCACTCCTT
**LC3**	CACTGCTCTGTCTTGTGTAGGTTG	TCGTTGTGCCTTTATTAGTGCATC
**MuRF1**	ACCTGCTGGTGGAAAACATC	ACCTGCTGGTGGAAAACATC
**MUSA1**	TCGTGGAATGGTAATCTTGC	CCTCCCGTTTCTCTATCACG
**MUSK1**	ATCACCACGCCTCTTGAAAC	TGTCTTCCACGCTCAGAATG
**Myogenin**	CCAACCCAGGAGATCATTTG	TCTGGGAAGGCAACAGACAT
**NCAM**	ACAATGCTGCGAACTAAGGA	TGCCACTTGACACAGGA
**Opa1**	ATACTGGGATCTGCTGTTGG	AAGTCAGGCACAATCCACTT
**p62**	CCCAGTGTCTTGGCATTCTT	AGGGAAAGCAGAGGAAGCTC
**Runx1**	CGGCAGAACTGAGAAATGCT	CAACTTGTGGCGGATTTGTA
**Smart1**	TCAATAACCTCAAGGCGTTC	GTTTTGCACACAAGCTCCA
**TNFα**	CACAAGATGCTGGGACAGT	TCCTTGATGGTGGTGCATGA

Table S1. List of primers used for quantitative PCR analysis

TABLE S2

Antibody	Customer	Dilution	Analysis
Mouse anti-Actin AC 40	Sigma A4700	1:10000	WB
Mouse anti-Drp1	BD 611738	1:1000	WB
Mouse anti-GAPDH	Abcam ab8245	1:10000	WB
Mouse anti-NCAM	Millipore AB5032	1:200	IF
Mouse anti-OPA1	BD 612606	1:1000	WB
Rabbit anti-LC3 B	Sigma L7543	1:1000	WB
Rabbit anti-P62	Sigma P0067	1:1000	WB
Rabbit anti-phospho-4EBP1 (Thr 37/46)	Cell Signaling #9459	1:1000	WB
Rabbit anti-phospho-Akt (Ser473)	Epitomics EP2109Y	1:1000	WB
Rabbit anti-phospho-S6	Cell Signaling #2215	1:1000	WB
Rabbit anti-total 4EBP1	Cell Signaling #9452	1:1000	WB
Rabbit anti-total Akt	Cell Signaling #9272	1:1000	WB
Rabbit anti-total S6	Cell Signaling #2217	1:1000	WB
Rabbit anti-VDAC	Cell Signaling sc-4866S	1:10000	WB
Rabbit anti-VDAC	Cell Signaling sc-4866S	1:100	IF
Total OXPHOS Rodent WB Antibody Cocktail	Abcam 110413	1:5000	WB

Table S2. Antibodies used in this study

TABLE S3

**F0 Cross**	**Genotype**	**Expected %**	**Observed %**	**χ^2 value**	**P value (df=3)**
♂ ($O^{fl/fl}$; $D^{+/+}$; Cre-) X ♀($O^{+/fl}$; $D^{+/fl}$;Cre+)	$O^{+/fl}$; $D^{+/+}$; Cre-	25	7,143	12.29	0.0065
	$O^{+/fl}$; $D^{+/+}$; Cre+	25	7,143		
	$O^{+/fl}$;$D^{+/fl}$; Cre-	25	64,29		
	$O^{+/fl}$;$D^{+/fl}$; Cre+	25	21,43		

TABLE S3

F1 Cross	Genotype	Expected %	Observed %	χ^2 value	P value (df=17)
♂($O^{+/fl}$; $D^{+/fl}$; Cre-) X ♀($O^{+/fl}$; $D^{+/fl}$; Cre+)	$O^{+/+}$; $D^{+/+}$; Cre-	3,558	1,299	58.33	0.0001
	$O^{+/+}$; $D^{+/+}$; Cre+	3,558	0		
	$O^{+/+}$; $D^{fl/fl}$; Cre-	3,558	9,091		
	$O^{+/+}$; $D^{fl/fl}$; Cre+	3,558	5,195		
	$O^{+/+}$; $D^{+/fl}$; Cre-	7,143	3,896		
	$O^{+/+}$; $D^{+/fl}$; Cre+	7,143	1,299		
	$O^{fl/fl}$; $D^{+/+}$; Cre-	3,558	9,091		
	$O^{fl/fl}$; $D^{+/+}$; Cre+	3,558	7,792		
	$O^{fl/fl;}$ $D^{fl/fl}$; Cre-	3,558	0		
	**$O^{fl/fl}$; $D^{fl/fl}$; Cre+**	**3,558**	**0**		
	$O^{fl/fl}$; $D^{+/fl}$; Cre-	3,558	2,597		
	$O^{fl/fl}$; $D^{+/fl}$; Cre+	3,558	3,896		
	$O^{+/fl}$; $D^{+/+}$; Cre-	7,143	2,597		
	$O^{+/fl}$; $D^{+/+}$; Cre-	7,143	7,792		
	$O^{+/fl}$; $D^{fl/fl}$; Cre-	7,143	1,299		
	$O^{+/fl}$; $D^{fl/fl}$; Cre+	7,143	1,299		
	$O^{+/fl}$; $D^{+/fl}$; Cre-	10,7	20,78		
	$O^{+/fl}$; $D^{+/fl}$; Cre+	10,7	22,08		

TABLE S3

F2 Cross	Genotype	Expected %	Observed %	χ^2 value	P value (df=11)
♂($O^{+/fl}$; $D^{fl/fl}$; Cre-) X ♀($O^{+/fl}$; $D^{+/fl}$; Cre+)	$O^{+/+}$; $D^{+/fl}$; Cre-	6,25	6,383	11.64	0.3916
	$O^{+/+}$; $D^{+/fl}$; Cre+	6,25	2,128		
	$O^{+/+}$; $D^{fl/fl}$; Cre-	6,25	6,383		
	$O^{+/+}$; $D^{fl/fl}$; Cre+	6,25	8,511		
	$O^{+/fl}$; $D^{+/fl}$; Cre-	12,5	17,02		
	$O^{+/fl}$; $D^{+/fl}$; Cre+	12,5	10,64		
	$O^{+/fl}$; $D^{fl/fl}$; Cre-	12,5	17,02		
	$O^{+/fl}$; $D^{fl/fl}$; Cre+	12,5	14,89		
	$O^{fl/fl}$; $D^{+/fl}$; Cre-	6,25	2,128		
	$O^{fl/fl}$; $D^{+/fl}$; Cre+	6,25	8,511		
	$O^{fl/fl}$; $D^{fl/fl}$; Cre-	6,25	6,383		
	**$O^{fl/fl}$; $D^{fl/fl}$; Cre+**	**6,25**	**0**		

F3 Cross	Genotype	Expected %	Observed %	χ^2 value	P value (df=7)
♂($O^{fl/fl}$; $D^{fl/fl}$; Cre-) X ♀($O^{+/fl}$; $D^{+/fl}$; Cre+)	$O^{fl/fl}$; $D^{+/fl}$; Cre-	12,5	13,89	11.56	0.1162
	$O^{fl/fl}$; $D^{+/fl}$; Cre+	12,5	11,11		
	$O^{fl/fl}$; $D^{fl/fl}$; Cre-	12,5	8,333		
	**$O^{fl/fl}$; $D^{fl/fl}$; Cre+**	**12,5**	**0**		
	$O^{+/fl}$; $D^{+/fl}$; Cre-	12,5	19,44		
	$O^{+/fl}$; $D^{+/fl}$; Cre+	12,5	8,333		
	$O^{+/fl}$; $D^{fl/fl}$; Cre-	12,5	13,89		
	$O^{+/fl}$; $D^{fl/fl}$; Cre+	12,5	25		

Table S3: Genotype distribution analysis in offspring. The comparison of the value predicted by Mendelian inheritance with the observed value shows the absence of homozygous mice with the double deletion of OPA1 and DRP1 (indicated in bold), and suggests that homozygous DKO mice died in utero.

TABLE S4

Phenotype/Signaling	HSA OPA1 (inducible) [1]	HSA DRP1 (inducible) [2]	HSA OPA1/DRP1 DKO (inducible)
**Muscle Atrophy**	Yes	Yes	Yes
**Myofiber Degeneration and Regeneration**	No	25%	6%
**Force drop/Weakness**	Yes	Yes	Yes
**Muscle Denervation**	Yes	Not done	Yes (early)<br>No (late)
**Mitochondrial morphology**	Fragmented	Elongated	Elongated/<br>Onion-like structures
**Mitochondria dysfunction**	Yes	Yes	Yes
**Mitophagy**	Not done	Impaired	Impaired
**Autophagy**	Increased	Impaired	Impaired
**ER stress (UPR)**	Yes	Yes	Yes
**Increased UPS**	Yes	Yes	Yes (early)<br>No (late)
**Oxidative Stress**	Yes	No	No
**Muscle FGF21**	Increased	Increased	Increased (early)<br>Normal (late)
**Serum FGF21**	Increased	Increased	Increased (early)<br>Normal (late)
**Glycemia**	Reduced	Reduced	Increased (early)<br>Normal (late)
**Systemic Inflammatory Response**	Yes	No	No
**Survival**	90-120d after treatment	Normal lifespan	Normal lifespan

TABLE S4. Comparison of the phenotype and signaling of muscle-specific single Opa1 and Drp1 knockout mice with DKO mice.

early:70 days after treatment, late:365 days after treatment

TABLE S5.

Phenotype/Signaling	HSA DRP1/OPA1 (DKO) muscles	Sarcopenic muscles
**Muscle loss**	Yes	Yes [3, 4]
**Force drop/Weakness**	Yes	Yes [3-5]
**Mitochondria Dysfunction**	Yes	Yes [6, 7]
**Mitochondrial Morphology**	Elongated	Elongated/Giant mitochondria [6, 7]
**ER stress (UPR)**	Yes	Yes [8]
**UPS**	Yes (early)<br>No (late)	Not induced [6]
**Autophagy**	Impaired	Impaired [6]
**Oxidative stress**	No	Yes [6, 7]
**FGF21**	Yes (early)<br>No (late)	Yes [9]
**Systemic Inflammatory Response**	No	Yes [3]
**Denervation**	Yes	Yes [3, 5, 6]

Table S5. Comparison between Inducible DKO and sarcopenic muscles.
early: 70 days after tamoxifen treatment, late: 365 days after tamoxifen treatment

References:

1. Tezze, C., et al., *Age-Associated Loss of OPA1 in Muscle Impacts Muscle Mass, Metabolic Homeostasis, Systemic Inflammation, and Epithelial Senescence.* Cell Metab, 2017.
2. Favaro G., R.V., Varanita T., Desbats MA, Morbidoni V., Tezze C., Albiero M., Canato M., Gherardi G., De Stefani D., Mammucari C., Blaauw B., Boncompagni S., Protasi F., Reggiani C., Scorrano L., Salviati L., Sandri M, *Drp1-mediated mitochondrial shape controls calcium homeostasis and muscle mass.* Nature Communications, 2019.
3. Ascenzi, F., et al., *Effects of IGF-1 isoforms on muscle growth and sarcopenia.* Aging Cell, 2019. **18**(3): p. e12954.
4. Sandri, M., et al., *Signalling pathways regulating muscle mass in ageing skeletal muscle: the role of the IGF1-Akt-mTOR-FoxO pathway.* Biogerontology, 2013. **14**(3): p. 303-23.
5. Carnio, S., et al., *Autophagy impairment in muscle induces neuromuscular junction degeneration and precocious aging.* Cell Rep, 2014. **8**(5): p. 1509-21.
6. Larsson, L., et al., *Sarcopenia: Aging-Related Loss of Muscle Mass and Function.* Physiol Rev, 2019. **99**(1): p. 427-511.
7. Romanello, V. and M. Sandri, *Mitochondrial Quality Control and Muscle Mass Maintenance.* Front Physiol, 2015. **6**: p. 422.
8. Demontis, F., et al., *Mechanisms of skeletal muscle aging: insights from Drosophila and mammalian models.* Dis Model Mech, 2013. **6**(6): p. 1339-52.
9. Conte, M., et al., *Human aging and longevity are characterized by high levels of mitokines.* J Gerontol A Biol Sci Med Sci, 2018.
